# Book Reviews

**Published:** 1808-06

**Authors:** 


### CRITICAL ANALYSIS
OF THE
### RECENT PUBLICATIONS
ON THE
### DIFFERENT BRANCHES OF PHYSIC, SURGERY, AND MEDICAL PHILOSOPHY.

---

### *The Edinburgh Medical and Surgical Journal, for April* 1808. *No. XIV.*

ARTICLE 1.—*Reports from the Select Committee of the House of Commons, appointed to enquire into the State of Lunatics. Ordered to be printed April* 15, 1807.

ARTICLE 2.—*Observations on the General Treatment of Lunatics, as a Branch of Medical Police.* By ANDREW DUNCAN, Jun. M. D. Professor of Medical Jurisprudence in the University of Edinburgh.

BY this paper, it appears that the act of parliament relative to commissioners licensing mad-houses, does not extend to Scotland. However we may regret the inconveniences our sister kingdom has sustained by this event; it may, at least, furnish a comparative view of the two countries, and, under the present inquiry, assist us in forming just conclusions.

ARTICLE 3.—*A Report on the Influence of a moist Atmosphere in aggravating the Form, and retarding the Cure of the Infectious Ophthalmia, drawn up by Desire of Deputy Inspector* FERGUSON. By JOHN VETCH, M. D. Assistant Surgeon with Ophthalmia Detachments, at Riding-Street Barracks, August 12, 1808.

IN this valuable paper, Dr. Vetch produces abundant proof from his own, and the observations of others, that humidity in any shape is highly injurious to those afflicted with Egyptian Ophthalmia; that in marshy situations the disease will assume the intermittent form, and even under the common circumstances of a clouded atmosphere or rainy season, that it will always be exasperated. It gives us much pleasure to find such attention paid to our brave defenders; and that as soon as the Inspector General was made acquainted with these facts, the Ophthalmia cases were removed from Riding-Street, to the Barracks at Selsea. The event has proved as Dr. Vetch expected, and as every one must wish.

ARTICLE 4.—*Remarks on the Difference between the infectious Ophthalmia and that produced by the artificial Application of irritating Substances to the Eyes.* By JOHN VETCH, M. D. Assistant Surgeon with Ophthalmia Detachments, at Riding-Street Barracks.

THIS paper contains many very useful diagnostic remarks, which we shall extract, that they may be as generally known as possible.

"To point out the difference between this factitious disease, and the infectious ophthalmia, which first appeared in the army during the Egyptian expedition, and in which it still prevails to a considerable extent, some leading facts are here briefly contrasted, in the hopes that they may be useful in assisting so necessary a discrimination, and to prevent, by a hasty belief, that there exists no other than a factitious ophthalmia, the dereliction of those measures which, after the loss of much time, have been found sufficient to arrest the progress of the natural disease.

"1. The affection of the eyes which prevailed in the 28th regiment of foot, and which has been proved in the greatest number of cases to have been caused by the wilful application of irritating substances to the eyes, was confined entirely to the privates and non-commissioned officers of the regiment.

"Where the infectious ophthalmia has prevailed, it has uniformly affected the women and children, in a proportion equal to that of the men: it has been frequently communicated to the officers, both military and medical. It has appeared among the inhabitants of the neighbourhood of the disease; it has also made its appearance among the children of the Royal Military Asylum, where for some time it prevailed to a considerable extent.

"2. The counterfeit ophthalmia continued to present itself after the adoption of the most vigorous measures against the action of contagion or infection.

"The progress of the infectious ophthalmia has been, in those instances with which I have been connected, effectually arrested by the removal of the affected, on the first appearance of the disease; their complete separation from the healthy as long as the slightest symptom remains; and the vigorous enforcement of every measure against the operation of infection.

"3. In the counterfeit opththalmia, the inflammation was, for the most part, confined to one eye, and that almost always the right.

"In above 1000 cases of the infectious ophthalmia, I have met with but a very small number, not exceeding six, where both eyes have not been affected.

"4. The counterfeit ophthalmia was sudden in its progress, having generally occurred, and arrived at its acme, in the course of the night.

"The infectious ophthalmia advances by a gradual and uniform progress after its first commencement.

"5. In the counterfeit ophthalmia, the swelling was chiefly in the conjunctiva, and was not attended with much purulency.

"The tumefaction of the palpebræ, and great purulent discharge, are particularly characteristic of the infectious ophthalmia.

"6. The artificial ophthalmia generally ceased as soon as the vision of the eye was rendered imperfect.

The infectious ophthalmia continues to harrass the patient for months months after the destruction of the eye, and the purulency and tendency to relapse often remain for years after the total loss of sight.

"7. As the counterfeit ophthalmia was generally confined to one eye, so was the loss of sight, a very small number of the 28th having lost both eyes.

"From the infectious ophthalmia, a greater number have lost both eyes than one.

"8. The eyes which were rendered unserviceable from the artificial ophthalmia, had seldom suffered much organic alteration.

"Among the eyes which have been lost from the natural ophthalmia, a few are merely in consequence of a cloudiness of the cornea, and which may yet recover; but in by far the greater number, the organ is completely deformed, either by the formation of staphyloma, the complete evacuation of the humours, and a consequent flattening of the eye, from obliteration of the pupil, and adhesion of the iris with the cornea, cataract, amaurosis, and pterygion."

Article 5.—*Remarks on* Mr. Ellis's *Theory of Respiration.*— By John Bostock, M. D. late President of the Medical Society, &c. &c.

It is with much pleasure we find the remarks of Mr. Ellis have excited the attention of that industrious experimenter and able physiologist, whose name appears at the head of this paper. Dr. Bostock's opinions are so well known, and so generally admitted, that we deem it unnecessary to give even the short recapitulation which this paper contains. We shall, therefore, at once, state his reply to Mr. Ellis's objections.

"I shall now, says Dr. B. consider the objections which Mr. Ellis offers to this hypothesis: He observes, that the air and the blood, while they are in the lungs, are separated, both by the membrane which forms the air cells, and by that which forms the coats of the vessels, and that the air must pass through both these bodies before it can act upon the blood. I may remark, in this place, that not only this argument of Mr. Ellis's, but some other parts of his reasoning, are founded upon the mistaken opinion, that *air* is supposed to enter the blood, and to exist in the vessels. This, however, is not the case; the oxygen on the one hand, before it enters the arterial, and the carbonic acid on the other, after it quits the venous blood, mutually give out and receive a portion of caloric, which is necessary to reduce each of these bodies to the gaseous state. Oxygen, however, is supposed to enter the blood, according to the hypothesis which I have undertaken to defend; and improbable as it might *a priori* appear, that it should have the power of penetrating a compact membranous body, the fact has been so well illustrated by the experiments of Dr. Priestley and others, that I cannot but consider it as one too well established to admit of any farther scepticism. He found that blood, when separated from the air by a bladder, was acted upon by it in the same manner as when no substance was interposed between, and that a thick stratum of serum also permitted the action of the air, although a thin layer of water, saliva, or oil, as effectually prevented it, as if they had been separated by a solid body. In what way the action takes place, whether it be in consequence of the mechanical structure of membranous matter, which permits the oxygen to pass through its pores, or whether it be owing to its affinity for oxygen, which causes it, as it were, to become saturated with this substance, before it transmits it, it is not our business at present to inquire; it is sufficient to state, that oxygen and blood can act upon each other, through a membrane, which is very much thicker, and probably much denser, than that which separates the blood in the rete mirabile of the lungs from the air in the bronchial cells.

"With respect to the actual change which takes place in air that has been exposed to the action of the blood, there can be no room for any difference of opinion; it is admitted on all hands, that it is diminished in bulk, that a part of its oxygen has disappeared, and that it has received the addition of a quantity of carbonic acid gas. The point in discussion is, to ascertain in what manner the carbonic acid gas is formed; and Mr. Ellis endeavours to disprove the former hypothesis, by showing that it cannot be derived from the venous blood, because there is no gas of any kind, and, of course, no carbonic acid gas existing in this fluid. But this objection, it is obvious, does not apply to my hypothesis. Mr. Ellis farther attempts to support his opinion, by showing that the carbonic acid produced in respiration is never formed, unless the blood has access to oxygenous gas, or to some gas containing oxygen.—The proofs which he brings of the fact are satisfactory, but they are as applicable to the hypothesis which he opposes, as to that which he supports. He has indeed omitted what may be considered as a more decisive argument against the existence of carbonic acid in the blood than any which he has adduced; viz. that the soda which is found in the serum, is in the pure or caustic state. I therefore agree with him in his conclusion, that the carbonic acid gas which is exhaled from the lungs, is formed in consequence of the decomposition of the atmospheric air that is received into them.

"A new point here comes to be considered: Mr. Ellis observes, that 'supposing the air to be thus decomposed by the blood, it still remains a question, whether it has been first attracted by that fluid, then decomposed, and afterwards in part expelled; or, whether the decomposition has been effected without such previous attraction and intermixture of air.' Now, the statement of the first part of this dilemma is not perfectly correct. It is not supposed that the air is absorbed by the blood, and afterwards in part expelled, but that a portion of its oxygen is attracted, which, after entering into a variety of new combinations, is only discharged as a constituent of some of these new compounds. This appears to be a sufficient reply to the only, or at least the principal objection which which Mr. Ellis offers to my hypothesis, that 'granting to the blood this power of attracting air, or its oxygenous portion, it is not easy to conceive why it should so readily lose it, and again give out this air in the form of carbonic acid.' The only part of the oxygen than can be supposed to undergo this rapid succession of changes, is the quantity which is necessary to convert the oxide of carbon into carbonic acid; and it is not at all essential to the hypothesis, that this portion of oxygen should enter into the blood. Mr. Ellis's observation, that the only argument in favour of the entrance of oxygen into the blood, is derived from the diminution in bulk which the air undergoes by the process of respiration, will, I think, be best answered when I come to state the positive arguments in favour of my hypothesis.

"Mr. Ellis again recurs to the objection that was noticed above, that the air and the blood are not in contact in the lungs, but that contact is necessary to the action of chemical affinity. Without entering into the general question, I think it sufficient once more to refer to Dr. Priestley's experiments, in order to prove that this action really does take place, under apparently less favourable circumstances. I am therefore obliged to dissent absolutely from the assertion of Mr. Ellis, that this supposition 'is wholly gratuitous, and in opposition to the results of direct experiments.' His remarks, that if air can penetrate the bronchial cells, it would, still more readily, pass into the cellular substance that connects these together, is incorrect, in the first place, in as much as it is not *air*, but *oxygen* which is supposed to pass into the blood-vessels; and, secondly, the passage of the oxygen only takes place because it is attracted by the blood. I may be permitted to pass over the arguments that are again adduced to prove that air does not enter the blood, or that it is not taken up by the absorbents, because neither of these suppositions enters into my hypothesis, nor do I think myself called upon to follow the author through his examination of Mr. Davy's arguments in favour of the absorption of air by the blood, because, however highly I may estimate the talents of this distinguished chemist, I have ventured to differ completely from him on the subject under discussion. It will also be at once obvious, that the next of Mr. Ellis's objections does not attach to my hypothesis. He conceives that the air cannot be absorbed by the blood, and undergo the decomposition and subsequent alteration that have been described, because the change which it experiences is so considerable, and the period of one act of respiration so short, that there would not be sufficient time to bring about all these supposed changes. The fact indeed is, that Mr. Ellis's hypothesis supposes all the changes to be effected in the lungs, whereas, according to my idea, the greatest part of them are produced in the course of the circulation.

"The observation that Mr. Ellis afterwards makes, in support of his hypothesis, that the formation of carbonic acid, and the abstraction of oxygen are always exactly proportional, is obviously

 as as favourable to my hypothesis as to his own; and the same remark may also be made respecting the fact which is brought forwards, that there is no production of carbonic acid when hydrogen is respired. What Mr. Ellis says respecting the dark colour of venous blood, is worthy of consideration; and it must be admitted, that we have no direct proof of its depending upon a superabundance of carbon in it. But it is, I apprehend, obvious, that as far as our opinions throw any light upon the subject, my hypothesis has the advantage. The change which the venous blood undergoes, according to Mr. Ellis, is simply the abstraction of carbon; whereas the other hypothesis supposes, that while carbon is removed, oxygen is received, so that the change of colour may be attributed to either or both of these circumstances."

On this difference of opinion, we shall only make one remark, which the reader will find contained in our analysis of Mr. Ellis's "Inquiry," namely, that we are by no means satisfied with the proofs that the blood attracts oxygen through the membrane of the pulmonary cells. Dr. Priestly shows that this may take place through a membrane much thicker than those cells; but this membrane was dead. Let us recollect, that during life, no change takes place in the purple appearance of the blood in the lips under temporary suffocation, or in paroxysms of angina pectoris, arising from any mal-formation in the heart. Yet, after death, the lips and cheeks recover their florid appearance. The reader will perceive, that our objection applies to Mr. Ellis's theory almost as much as to Dr. Bostock's hypothesis; for it is not to be imagined, that after death, any thing but chemical changes can take place: consequently, the alteration in the colour of the blood cannot be imputed to any excretion from it after death, any more than to mere chemical attraction during life. We have offered the above solitary remark, rather to show our impartiality, than from any wish to engage in the contest. We are aware that the subject requires experiments which we have not leisure to pursue, and that it is easier to object to the conclusions of others, than to form any of our own. We wish our readers to feel with us highly grateful for the labours of these ingenious gentlemen, trusting they will continue to inform us, and receive our doubts as we mean to express them.

ARTICLE 6.—*The following History of a Case of Hysteralgia was read at a Meeting of the Medical Society of Liverpool.* By JOHN RUTTER, M. D. Physician of Liverpool.

THIS case, had it been related with more minuteness, might have afforded many practical remarks. The symptoms during life were such as might have puzzled the ablest physicians, and can never be well understood but by an accumulation of well recorded symptoms with subsequent dissections. They would, generally speaking, have been considered as nervous or hysterical. Thirty-six years before death, a tumour which had been for some years forming, seemed to burst internally, and discharge its contents by the rectum.

rectum. This discharge gradually assumed the appearance of healthy pus, and the parts healed. But from that time the patient complained of incessant pain, as she described it, in the womb, and had all those dreary symptoms which attend a constitution perpetually harrassed by an incurable complaint. At the age of 77 she died; and the following is the account of the dissection, by Mr. Christian and Mr. Hay, with Dr. Rutter's remarks.

"The external parts of the body presented nothing very remarkable, except a tumour in the right hypogastrium, about the size of an egg, and situated under the integuments, or rather the abdominal parietes. The labia externa, and also the skin about the anus were in a state of incipient gangrene.

"Upon opening the abdomen, the parts in general appeared healthy, and in their natural situations. The colon, however, contained large proportions of scybala, more especially that part of it called the cæcum, which was completely distended with those substances, and, by its weight, or from other causes, had descended into the pelvis. The tumour above noticed, as appearing through the integuments, was also a portion of the same substance.

"The other viscera contained in the pelvis were not in a natural state. The uterus, and that part of the colon which terminates in the rectum, were firmly attached even to the very fundus, immediately above which, and situated upon the anterior part of the gut, was a tubercle of a glandular-like structure, and resembling a small raspberry, but which was hard in its texture, approaching to a state of scirrhosity; yet its connection with the intestine was neither very intimate nor firm. Upon separating the uterus from its connection with the intestine, which required a considerable degree of force, a cyst was opened, which was found to contain a blackish bituminous-like substance, of the consistence of a thin extract, and about two drachms in quantity. Upon tracing the cyst, it was found to extend downwards about two inches, independently of a sinus at its inferior extremity about two inches more, running towards the left side of the rectum, and piercing its peritoneal and muscular coats; but no opening could be discovered into the rectum, either at this or any other part. The upper part of this cyst was firmly attached to the tubercle above described; but it appeared to have no particular connection with the uterus, which was extremely small, and perfectly soft in its substance, presenting no marks whatever of disease, except a slight vesicular appearance on its internal surface, but which did not extend to the vagina.

"The bladder was but slightly distended, and felt somewhat firmer than usual, being somewhat thickened in its substance; and the internal membrane exhibiting a rough surface, as if corrugated by the application of an astringent substance; but no appearance of inflammation existed in this part, nor even at the neck of this viscus, although a slight degree of it was observed in the urethra, about half an inch from its extremity.

"The rectum, throughout its whole extent, was considerably distended with fæces; the circumference in general being about six inches, except at the upper part of the pelvis, where it would only receive the end of the finger.

"The ureters and kidneys were free from disease, and the rest of the viscera appeared healthy. The spleen was unusually small, and the gall-bladder was filled with a biliary concretion, except at that end which terminates in the ductus cysticus, where about two or three drachms of thick bile were collected.

"The aorta was completely ossified above its division into the iliacs; and the same state of the coats of the vessel extended upwards of two inches, and existed also in some of the vessels within the pelvis."

"To this report it may be added, that the ovaria and Fallopian tubes were perfectly free from disease; and that no appearances were found, which could in the least account for the continual sense of heat and soreness, immediately under the abdominal parietes, in that part where the abscess had protruded.

"Upon comparing the symptoms with the appearances just related, it will at once be perceived, that the examination has thrown scarcely any light on the nature and cause of the pain always referred to, and always supposed to have had its seat in the uterus. It is not easy to believe that the vesicular appearance described on the internal surface of the uterus, could have occasioned such severe and long continued torment. And no other change was observed in the structure of the uterus, no thickening, no hardness, no scirrhosity, no ulceration, nothing which affords any explanation of this distressing symptom. I think there could be no deception in the feelings of the patient for so long a period. The pain was constantly fixed in one place, and that place was always believed by herself to be the uterus. There does not seem to be any thing in the other appearances observed, which would lead to a suspicion that she was mistaken in this belief. For it is not probable, that the coats of the cyst were endued with a degree of sensibility sufficient to have given occasion to such suffering. And it does not appear that the bituminous-like substance found in it had, by any of its properties, chemical or mechanical, produced any effect whatever on the coats of the cyst. That substance seems to have been altogether inert; for if either inflammation, erosion, or ulceration had been occasioned by it, they would not have escaped the observation of the gentlemen before mentioned, who examined the parts with great care. But even if such effects had been detected, still it is highly improbable, that the pain excited by them should have been as severe as that which I have described, or continued as long.

"It may, however, be supposed, that the pain may have been occasioned by the tubercle. Such a supposition must be founded upon an opinion that the tubercle was of a scirrhous nature. Now, scirrhous scirrhous tumours are characterised by their hardness, their indolence, the slowness of their growth, and by their not changing the natural colour of the part. When a scirrhous tumour is approaching to the state of carcinoma, it becomes painful, but the pain is always more or less lancinating. At the same time, the scirrhus enlarges more rapidly than before, and changes its form, and becomes unequal, pointed, and angular. But the tubercle above mentioned had not undergone this change. It was small, round, and circumscribed; hard, firm, and of the natural colour; and, therefore, a strong presumption arises, not only that it was not the cause of the pain before described, but that it was not painful at all. It does not appear to be consistent with the observations which have hitherto been made of the progressive changes of scirrhus into cancer, to suppose that a scirrhous tumour could have excited, for such a length of time, such 'horrid pain;' for such was the epithet by which this patient described the pain, without undergoing great changes, and producing very extensive destruction in all the neighbouring parts.

"There seems, then, to be little or no reason to conclude, that she was mistaken in her belief with respect to the seat of the pain; and if so, it is an extraordinary circumstance that the pain should have continued in the uterus, without one day's cessation, for 26 years, and that no derangement in the structure of that organ should be discovered after death, sufficient to account for it.

"If this pain was not occasioned by an organic affection of the uterus, to what cause is it to be ascribed? On this point, perhaps, different opinions may be entertained. It appears most probable to me, that this symptom was hysterical. The constitution of this patient was disposed to hysterical affections. Many of her symptoms were entirely of that description. The violent eructions, the depression of spirits, the faintness, the frequent discharge of pale coloured urine, the palpitation of the heart, the flutter and agitation which accompanied the pain, and her constant proneness to forebode the worst termination of her complaints, were all hysterical. All these symptoms increased with the increase of the pain, and declined as it declined. As they were constantly associated together, it is probable that they were respectively symptoms of one general affection, and that affection was hysteria. This opinion is supported by other considerations. There was originally in her constitution that degree of sensibility which is favourable to the production of such a disease, and which had no doubt been greatly increased by the sedentary habits which she had been constrained to adopt during the greatest part of her life. And the dependence of the pain upon this constitutional sensibility, appears to me to be evinced by the circumstances already mentioned, of the pain having been greatly mitigated, and the other hysterical symptoms having been greatly lessened, whilst the paralytic affection remained, and of their return with their former former violence, when the sensibility of the system was restored to its former state.

"There is something singular in the regular exacerbations of the pain every day, as well as in those which were observed at the lunar periods. I know no explanation which can be given of this. Dr. Whytt, in one case for which he was consulted, inferred from periodic pains in the hypogastric region, the existence of scirrhus in the uterus beginning to turn cancerous. The case in a few circumstances bears a distant resemblance to the case before related. The following are his words: 'As I was only consulted for this person at a distance, I never learned whether her body was opened or not, but I think there can be little doubt, that almost all her complaints, and particularly the sharp periodic pains in the hypogastric region, were owing to a scirrhus in the uterus beginning to turn cancerous.' The present case, however, shews, incontestibly, that periodic pains in the hypogastric region cannot be relied upon as proofs of the existence of scirrhus in the uterus about to degenerate into cancer.

"With respect to the reason of the other morbid appearances observed in the contents of the pelvis, a few words may suffice. It seems in the highest degree probable, that the cyst found between the uterus and rectum was the remains of the abscess, and that its firm adhesion to these viscera had been occasioned by the inflammation which must have attended it in its progress. It is equally probable, that the sinus at the bottom of the cyst, and which was found to penetrate the peritoneal and muscular coats of the rectum, was the channel by which the contents of the abscess were discharged into that bowel, although the opening into it appears to have been obliterated. So far these phenomena seem to be easily explained. But there is considerable difficulty in determining the nature and origin of the dark coloured substance found in the cyst. It seems, *prima facie*, to have been the remains of blood poured into the cyst, of which the thinner parts had been absorbed. I mention this rather as a conjecture, than as an opinion.

"After all that has been said, it must be admitted, that some of the circumstances of this case are uncommonly obscure; and if the explanation which I have attempted to give of the leading symptom be not the most probable and rational, I know no other which can be given of it."

In reading the above remarks, we cannot withhold our surprize that so little notice is taken of the state of the colon and cæcum, so distended with scybala, that by weight and other causes, the cæcum had descended into the pelvis. Such an unnatural state of parts immediately and constantly engaged in important offices, must have produced constant inconveniences, the nature of which would, in a great degree, depend on the peculiar constitution of the patient. But we forbear to dwell any longer on the subject, lest we should render any of our readers backward in recording valuable ble cases with dissections. Dr. Rutter's paper is highly important, and we trust he will consider our remarks in no other light than as conjectures, which might have been laid aside, had we possessed all his knowledge of the case.

ARTICLE 7.—*Case in which the Cæsarean Operation was successfully performed twice on the same Woman. Communicated by* Dr. CHISHOLM.

THIS case is so curious, as well as important, that we cannot withhold it from our readers.

" In October, 1805, Monsieur D'Ariste, an eminent surgeon accoucheur in the island of Martinique, was employed to attend a first labour, of which the following particulars are the most interesting: The lady was 25 years of age, three feet ten inches high, of a very ricketty constitution, and much deformed. During her pregnancy, nothing remarkable occurred; but to the period of delivery, her friends looked forward with a degree of anxiety for which they had but too much cause. The surgeon, sensible of the disadvantages opposed to him, proceeded with much caution. The touch increased his apprehensions, as it convinced him that a living child could not pass through the natural passages, the diameter from the pubes to the sacrum being under two inches. The patient was sensible of her danger, but she had good spirits and much fortitude; the child also was lively. So circumstanced, and it being late in the evening, M. D'Ariste thought it prudent not to proceed to extremities till next morning, when he could have the assistance of his professional friends. At that time, accordingly, being joined by his brother and others, and the case being maturely considered, their united opinions were in favour of the Cæsarean operation, in preference to the certain destruction of the child, by the scissars and crotchet. The patient consented readily; and being properly placed on a mattrass, raised on a table, her legs were secured by assistants, and an attempt was made to empty the bladder, but from the pressure of the uterus, the catheter could not be introduced. A straight incision was now made along the *linea alba*, beginning an inch below the umbilicus, and extending to nearly the same distance from the pubis; several arteries were divided, and immediately secured by ligatures; a portion of the intestines were protruded at the wound, but easily reduced; an incision was now made through the anterior part of the uterus, of the same extent as the external one; the placenta immediately presented itself, and was separated. The surgeon then introduced his hand in search of the child's feet, and having laid hold of them, had little difficulty in completing the delivery. As soon as the secundines were brought away, the natural contraction of the uterus restrained the hæmorrhage, which was at first considerable. The edges of the external wound were brought together by sutures, and simple dressings were applied, and retained by a proper bandage round the body. The wound was dressed daily, and in three or four weeks the patient was able to to sit up in a chair, only prevented from stirring about by the debility consequent to so formidable an operation. Some untoward circumstances attended the progress of the cure; a diarrhœa was at times troublesome, and she had some accessions of fever, all of which, however, yielded to common remedies. The child was weak and faint for a few minutes after it was brought to light, but restored by the common cordials; nothing particular was noticed with respect to it afterwards.

"The case appeared of such consequence to the Captain-General, Villaret Joyeuse, that he ordered an account of it to be published at the government press, which was accordingly done.

"In June, 1807, a gentleman of veracity, from Martinique, informed me, that the same woman had again been pregnant, and again Monsieur D'Ariste had performed the Cæsarean operation on her with the same success. I do not vouch for the truth of the second statement, but I believe it myself, from the character of my informant."

By this history, it appears that if the courage of French men is mere boasting, the women may be heroines, and also that the passion fof the other sex is at least mutual.

ARTICLE 8.—*Case of Chronic Rheumatism, and Observations on the Exhibition of Arsenic in the protracted Form of that Disease.* By GEORGE KELLIE, M. D. of Leith.

THE only thing remarkable in this paper is, that it obliges us again to observe how unjustly Mr. Jenkinson is deprived of the credit of having noticed this remedy in our Journal, before Dr. Bardsley's publication.

ARTICLE 9.—*Case of Jaundice, occasioned by the Pressure of a large Hydatid in the Liver, situated in the Region of the Vena Portarum.* By ANDREW DUNCAN, sen. M. D. Professor of the Institutes of Medicine in the University of Edinburgh.

ARTICLE 10.—*Biographical Sketches of* Dr. WILLIS.

THIS sketch, as it is very justly called, contains nothing particularly interesting that is not generally known.

The 13th Number of the Enquirer contains a string of questions which may be debated in a medical disputing Society with great advantage to the lungs of some, and the patience of others.

---

*Hints for the Consideration of Parliament, in a Letter to Dr.* JENNER, *on the supposed Failure of Vaccination at Ringwood; on the prevalent Abuse of variolous Inoculation, and on the Practice of the Small-pox Hospital.* By WM. BLAIR, Surgeon of the Lock Hospital, &c. &c. 8vo. p.p. 300. London, 1808.

AS we have ever been, and ever will be, while the evidence stands on its present basis, firm and zealous supporters of Vaccination, we shall not neglect any work, which has for its object, the promotion of that philanthropic practice. The present volume appears pears to us well calculated to promote Vaccination, by exposing and refuting the falshood and misrepresentation which have been industriously employed to prejudice and poison the public opinion respecting that invaluable discovery. We shall let the Author state his own views and objects.

"My first design in writing these pages was, to expose the unfair and mischievous conduct of an anonymous author, who had published a false account of the supposed failures of Vaccination at Ringwood, in one of our most popular Journals. My second motive was, to communicate such information relative to these numerous cases, as could not properly be given by the Directors of the Royal Jennerian Society, and yet might be expected from an individual in my situation.

"When I had printed above thirty pages, a fresh scene opened to my view: for, I discovered that the attack which had been recently made on vaccination was not of an ordinary kind, nor by a common or insignificant hand; but that it had been contrived and conducted with so much subtilty, perseverance, and effect, as to have awakened the public attention, and most shamefully deceived those who enquired after the real facts.

"Several popular engines were employed by the same person, at the same time, to accomplish his evident object; a serious pamphlet; a satirical paper; a vulgar placard; a ludicrous hand-bill; and various devices for the circulation of each, combined to excite a general and serious alarm! The ultimate and avowed intention of all this was, to call for the interference of the Legislature, and to hold up the late Report of the Royal College of Physicians to universal contempt.

"When I had nearly finished my observations upon this unprecedented misconduct in an *Hospital-Surgeon*, another subject commanded my attention. A truly respectable and well-known gentleman hinted to me, that some Members in both Houses of Parliament had listened to a single example of disaster, stated to have arisen from the practice of inoculating OUT-PATIENTS at the Small-pox Hospital. It struck my mind forcibly, that if an interest had been so easily excited, and a degree of jealousy raised by *one case*, it must be still more important to disclose the whole system now pursued at that Institution. I therefore immediately resolved to publish a full and faithful comment on the assertion which had been made by Mr. Birch, that "*the Small-pox Hospital was converted into a Cow-pox Station.*"

"In the course of my remarks, a most deplorable picture is given of the present horrid management of that charity, and the total dereliction of its former beneficial purposes! This portion of my volume, in particular, is that which I humbly submit to the candid consideration of those who alone can effectually remedy so great a national evil. It may appear to have been almost presumptuous in so obscure an individual as myself, to offer any hints to the highest Tribunal in the kingdom. But, when I remembered how how many great events have originated from the most inadequate beginnings, and how momentous this subject was to the whole nation, I could not refrain from making an honest attempt to direct the eye of men in power towards so extensive a source of calamity! Perhaps too, I might be encouraged by the hope, that ere long some patriotic Member of Parliament would deliberately consider the Answer received to a late Inquiry, made in compliance with an Address to His Majesty from the House of Commons. What causes impede the practice of vaccine inoculation in the United Kingdom? Is it of any benefit to have assigned those causes, if no strenuous effort be made for their removal? Ten years have nearly elapsed since vaccination began in England; and yet, it is so far from being now generally adopted, that our unwise countrymen, especially in London, discourage its use, and still spread the variolous pestilence, even more actively and widely than they did before Parliament had instituted this important inquiry!

"The College of Physicians" (as they said twelve months ago) 'conceive that the public may reasonably look forward with some degree of hope to the time when all opposition shall cease, and the general concurrence of mankind shall at length be able to put an end to the ravages at least, if not to the existence of the Small-pox.' This most desirable consummation, I fear, is not very likely to happen in the present age; and it certainly can never be reasonably expected, while one of our Hospitals is allowed to pour forth, year by year, several thousand infected patients into the heart of the British metropolis!

"A Member of Parliament, in the debate on Dr. Jenner's reward, very justly observed, that 'this country was in the singular situation of having discovered an antidote against the poison of the small-pox, and yet is the last to have so used that antidote as to put an end to the sad effects of the poison.' Another Honourable Member truly declared, 'that the benefits of this invention have been more extensively felt in every other country than in our own, which has given it birth; for, in other countries, the disorder of the small-pox has been already exterminated.' A third Member said, that 'if we were to prescribe a mode of spreading the contagion of small-pox, it would be difficult for human ingenuity to devise any thing better adapted for that purpose than to inoculate out-patients at the Small-pox Hospital, to the amount of two thousand in a year, and for these out-patients to resort there twice a week to be inspected.' It was also remarked by another Member, 'that this charitable establishment may have been a *pest* instead of a benefit to mankind, multiplying the number of victims, and creating the disease where perhaps it would not otherwise have existed.' Of this melancholy fact, I have given abundant proofs in the following pages.

"It will be learnt, with astonishment, that *four thousand five hundred and ninety-four* persons were inoculated at the Hospital in the year 1807, a number sufficient to disseminate the variolous

PLAGUE throughout all the world! This unprecedented exertion to diffuse the disease has occurred too, after a worthy Governor of that Institution had expressed his belief, in the House of Commons, 'that the Directors of the Small-pox Hospital do not now practice any but the vaccine inoculation; and if they do, it is a proper subject for legislative authority.' Another patriotic Member suggested, that the public ought to be 'secured against the effects of this contagion, in the same manner as is done in the case of the plague.' In reply to which, it was admitted by one of the then Secretaries of State, that 'the Legislature of any country may be well entitled to adopt compulsory measures, to prevent contagious maladies from spreading.' And the present Chancellor of the Exchequer acknowledged, 'that it is our duty to preserve life; and that the preservation of life included a care to prevent, as much as possible, those diseases by which that life is shortened.'

"Will not these Guardians of the public welfare now see it is high time to exercise that great national duty, of protecting us against a dreadful 'pestilence which walketh by noon day,' and ANNUALLY HAS DESTROYED ABOUT FIFTY THOUSAND BRITISH SUBJECTS? The multiform and unwearied efforts which a few individuals are daily making to propagate this destructive pestilence, can be successfully opposed in no other way than by the aid of wise, humane, and restrictive laws."

The insiduous and disingenuous conduct of Mr. Birch is very properly and compleatly exposed by a simple statement of facts. In these times, when able subjects are peculiarly wanted in the united kingdom, Mr. Birch says, 'in the populous part of the metropolis, where the abundance of children exceeds the means of providing food and raiment for them, this *pestilential* disease (small-pox) is considered as a *merciful provision* on the part of Providence, to lessen the burdens of a poor man's family.' If this be the ground on which the inveterate enemies of Vaccination oppose that mild and safe preservative, let them continue their opposition, and let the public judge of their motives.

When Mr. B. had taken laudable pains to inform Mr. Birch of the falshood of the statement made by him in the public papers respecting the Ringwood cases, and had also applied to his printer to have him properly informed on the subject, Mr. B. sent the following note to Mr. Birch.

"*Great Russel Street, Bloomsbury Square, Jan.* 19, 1808.

"Mr. Blair's compliments to Mr. Birch, and thanks him for the new pamphlet on vaccination, which he has received through the hands of Mr. Hughes, the publisher; but Mr. Blair is very sorry that the cause of truth needs a much more correct account of the affair at Ringwood, than Mr. Birch has therein given; and that the positive refusal of Mr. Birch to admit *authentic intelligence* on that subject, constrains Mr. Blair to expose such misconduct in a separate publication. Besides, if Mr. Birch had been sincere in his his inquiries after truth, he could not have brought forward the testimony of Mr. Westcott *against the cow-pox*, after the information sent him by that gentleman, about the end of December, and which Mr. Birch has entirely suppressed!

"*To* JOHN BIRCH, Esq.
*Spring Gardens*."

Mr. B. afterwards proceeds to prove by *direct evidence*, that Mr. Birch is the author of the anonymous publication respecting the Ringwood Cases; and that he published and asserted a statement of them to be true, which he *knew to be false*.

When we consider the very extraordinary conduct of Mr. Birch, we are disposed, with our author, to think the most charitable supposition is that of insanity.

"There is," says Mr. B. "one very striking fact demonstrated by the contents of Mr. Birch's anonymous Narrative, too much affecting the honour and dignity of the Royal College of Surgeons, to be passed over without comment: I mean, his having access to the private papers or books, and the College 'REGISTER' (as he names it); AND HAVING THENCE EXTRACTED AND PRINTED WHATEVER HE PLEASED, FOR THE PURPOSE OF CALUMNIATING THE ROYAL COLLEGE OF PHYSICIANS, AND OF EXPOSING THEIR REPORT ON VACCINATION TO PUBLIC CONTEMPT. Has he not indeed done this? If he be the author of the vile pamphlet in question (which I think clearly proved) he fraudulently availed himself of his opportunity and privilege, as belonging to the Court of Assistants: But, why do I say *privilege;* when I know and affirm, that no individual, *even a Member of that Court*, has a right to examine or touch those papers, without the express leave of the Board of Curators? I have authority to declare, that Mr. Birch had no such permission; that he did not condescend to ask for it; and that he acted thus treacherously, without the knowledge of the Secretary himself! What will now be done with this 'HONEST MAN,' I pretend not to foretell; but I am persuaded the Curators will do justice to themselves, to both Colleges, and to the World at large.

"I by chance discovered the congratulatory letter which you sent to me when Mr. LIPSCOMBE's party was defeated, at the great debating-room in Piccadilly, during your last visit to London; but, alas! how mistaken you were, Sir, in supposing '*that would be the expiring effort of anti-vaccinists. They die hard,*' you write, '*and hardened.*' That they are '*hardened*' enough, I admit; but not that they are *dead*. You have already had sufficient evidence of this; and I confess they appear even still more 'hardened' than you suspected, during what seemed to be their 'expiring' moments. In my former 'DIALOGIC PAMPHLET,' as Dr. Lettsom calls it, I shewed to what lengths this 'chosen band' had then gone in their mad career: but the late conduct of Mr. Birch really seems to be a full consummation of all the stratagems they could invent, as a *dernier resort!*"

The

The whole of Mr. B's attack and exposure of Mr. Birch's falsehoods, perversions of fact, and wilful blindness to the light of truth, has our entire approbation. Our author is not satisfied with disarming his adversary and vanquishing him, but he diminishes his own triumph by making his antagonist contemptible.— He represents him as often appearing in public with black eyes, as being fond of Burgundy and Champaigne. We, on the contrary, are of opinion, that Mr. Birch is never more *innocently* employed than when behind the scenes at the opera, or when quaffing Champaigne and Burgundy. On all occasions, and under all circumstances, we think that the private follies, or even vices of any individual, have nothing to do with the discussion of his book or opinions.

When Mr. B. has finished his answer to Mr. Birch, he proceeds to some other points, which he thinks his present publication ought to notice. The first of these is the London Vaccine Institution, established by a great number of persons, who felt themselves treated with injustice and contumely by the Royal Jennerian Society. The number of persons vaccinated by the London Institution, and the charges of virus distributed from it, have far exceeded those of both the other Vaccine Institutions during the same period. The conduct of Dr. Walker, in endeavouring to impede persons going to the Royal Jennerian Society to be vaccinated, is as much reprobated by the supporters of the London Vaccine Institution as by Mr. B. himself. But Mr. B. has studied Mr. Birch's style and manners so long that he has made it his own. He thus speaks of the founders of the London Institution as a body: "There is abundant reason to suspect that many very excellent and benevolent persons, both medical and unprofessional, have been duped by the artful contrivances of a few disappointed and revengeful individuals, to countenance proceedings too disgraceful to be endured; proceedings, as you know, conceived in malignity, and accomplished by the grossest impositions! Our only comfort is, that some remote benefit may eventually arise, from transactions so little intended for the public good, and so obviously occasioned by a spirit of opposition to YOU, (Dr. Jenner)." We believe that there is not a passage in Birch, Rowley, or Moseley, the rancour, virulence, and falshood of which exceed the above.

The next collateral subject which Mr. B. proceeds to discuss is, the Small-pox Hospital. He makes a longer apology for touching upon the subject, than we should have thought necessary, considering the many objections which have recently and generally been urged against the practice of it.

"I should (says Mr. B.) have been gladly excused from offering my sentiments on so delicate an affair; but Salus Populi suprema Lex esto. I cannot be wholly excused in foro conscientiæ; and therefore must perform a task as unpleasant to my feelings as a surgical operation, &c. &c." And afterwards, "If you admit (as

I am sure you will) that I perform this task most reluctantly, you also must acknowledge that when a grievous abuse exists in a public institution, and duty calls for my assistance, I am not apt to be the last in setting a shoulder to the wheel."

We are not less struck with the correctness and propriety of the former part of this passage than we are with the self-importance assumed in the conclusion. From such an introduction, however, we are led to suppose, that the opinions and conduct of the Physician to the Small-pox Hospital will be treated with as much candour and tenderness as the imperious dictates of a public duty will permit.

Mr. B. very properly commences his inquiry by establishing the extent of the evil complained of, by the following authentic document from Mr. Highmore, the secretary.

*Patients during the Year* 1807.

| | | |
|---|---|---|
| Natural Small-pox, - - - | In | 170 |
| | Out | 37 |
| Inoculated - - - - - - - | In | 348 |
| | Out | 4246 |
| Vaccinated - - - - - - - | In | 44 |
| | Out | 1577 |
| In and Out Patients | | 6422 |

"Thus, Sir, it appears that 37 *out-patients* with the casual Small-pox, and 4246 inoculated, making in all 4283 out-patients, have been distributed through the streets of London and the neighbouring villages, during the last year!

The obvious inferences, deducible from the above statement, can require no illustration from us, and we entertain no doubt that a great majority of the public, who read Mr. B's reasoning and conclusions will agree with him and us, in condemning the conduct pursued at the Small-pox Hospital. If Mr. B. had confined himself to the subject of his book, and to the principal question at issue, he would have received our unqualified approbation; but at page 267 he has descended to a most shameful *personal* attack upon Dr. Adams, and such an one that would, if possible, be a disgrace to an antivaccinist. What has Dr. Adams's house, carriage, or his M. D. to do with Cow-pox protection?

If Mr. B. had not disgraced himself by this unpardonable outrage upon private feelings, and had omitted the falsehoods respecting the Subscribers to the London Vaccine Institution, we should have said that his book was well calculated to establish Vaccine Protection on an immoveable basis.

---

*An*

*An Attempt at a Systematic Reform of the Modern Practice of Adhesion; especially in relation to the Use and Abuse of the Thread Suture: with a View of its Merits comparatively with those of the Adhesive Strap: In the Surgery of Wounds: Being the Substance of a Course of Lectures intended to have been addressed to Students in Surgery.* By SAMUEL YOUNG, of the London College of Surgeons. Author of "*An Inquiry into the Nature and Action of Cancer, &c. &c.* 4to. London, 1808.

To this Work is prefixed an Advertisement, the object of which seems principally to show, how much the doctrines of Mr. Hunter on this important subject have, as in most others, been unattended to. Some preliminary remarks follow, on the importance of the doctrine, and the improvement surgery has received from a more accurate knowledge of the science of adhesion; the success that attended the practice of the early untaught surgeons, and the happiness of those since that time, who, when wounded, had no regular assistance to interrupt the natural progress of cure by adhesion. The first systematic introduction of the doctrine and mode of cure, by Mr. Hunter, and the pert objections to it, are humourously enough described.

"But the process of Adhesion was immutable, and could not be destroyed by human device. Its effects might be frustrated, but its nature could never be changed. It pursued its course unaltered, though persecuted, and established itself at last, not by persuasion, but conviction. I say by conviction, for the persecution was kept up to the very last. Animal adhesion owes nothing for its establishment to the impartiality of the world. The bigots to art were not inclined to let it have an easy conquest. We therefore find them obstinately disputing its establishment inch by inch. Even the labours of Mr. Hunter, who justly may be termed the founder of the *science* of adhesion, did not escape the caricaturists of Nature. This great surgeon, and physiologist, assisted by the London School, arranged and confirmed by experiments, facts that had hitherto been doubtful; and added many others to the catalogue which clearly established the process as an invariable act, covenanted by Nature herself. It is easily to be conceived that such proceedings would inevitably secure the patronage of Abuse. Abuse is the common lot of all great discoverers. The daring Harvey had this tribute paid him; and it would be idle to suppose that Mr. Hunter, engaged as he was, could escape a *common* fate. We therefore find a formidable clamour of *words* opened against him. The well-known fact of making a human tooth (recently extracted) adhere to an incision in a cock's comb, was held up as an abominable imposition, and turned into a ridiculous merriment. Such is the facility and impudence of artificial minds. They laugh at every thing that does not please their fancies, without troubling themselves whether the thing be true or not: A scientific investigation into the Laws of Nature is

 beneath beneath their art, or, more properly speaking, out of the reach of their comprehensions; and such is the force and facination of ridicule, that even the dramatic wit of Mr. John Bell, for a while, got the better of his excellent understanding, and he, among the rest, could not refrain from *pecking* at Mr. Hunter's cock's comb! He treated the fact as marvellous, and called it a Taliacotian tale. In his great work upon the Principles of Surgery, this Gentleman, however, has endeavoured to make his peace with Nature. In a note added to that work, he professes to be very happy to retract his former slanders. And since Mr. Bell is converted and become a true believer, it would be unfair to hint at his former heresy, any further than to show how futile and vain it is to oppose those truths which Nature recognizes. Hence we find that all opposition to the doctrine has only served to draw forth and establish the important realities of adhesion; and it is now received and acknowledged as one of Nature's best gifts! A kind of revealed physiology, which teaches us, above all other things, the true charity of surgery. And such are its beneficial results, that its establishment forms as great an epoch in the history of the healing art, as that of the circulation of the blood. Nor has the establishment of the one, effected less revolution than that of the other. The pompous monuments of art fell equally before them, and now lie buried in the ruins of their own confusion. In both discoveries Nature equally triumphed—in both, her great attribute is *simplicity*."

The writer proceeds with much severity to animadvert on the impropriety of sutures, excepting under certain circumstances, which are to be afterwards explained. He anticipates us in one observation, which has occurred to us in the perusal of almost every page, namely, that the practice of stitching is become quite obsolete, excepting under very peculiar circumstances; and we heartily participate with him in his surrpise, that writers of so late a date as Messrs. Benjamin and John Bell should have sanctioned such a practice. Still more were we astonished to find, that the latter, with his usual boldnes,s should give as a law, " Except in those cuts which are so slight as only to require a cloth to be wrapped about the part, *every wound ought to be sewed*." We cannot think such a rule will be followed by an apprentice of two years standing. The only aprehension we feel is, lest this should be considered on the Continent as the present state of *British Surgery*.

The work begins with a somewhat tedious defence of the term adhesive inflammation, against Mr. Bell, who in his usual dashing manner, we are told, objects to it, saying, " that it is no better than to call a cure a disease." We congratulate our Author on the patient manner in which he takes up the question, if we can call it such, when the whole turns upon a well known fact, that many diseases are cured by inflammation; and also, that in many instances inflammation is not only an healthy but an absolutely necessary process. In order, however, to complete his work the more more systematically, a reference is made to what we conceive the Brunonian System, excepting that we have the word irritability instead of excitability. If in this instance we are mistaken, the Author will pardon our ignorance. We are ready to admit that the passage contains some useful remarks, but without transcribing the whole, which would exceed our limits, it would be unintelligible. At the conclusion, we have the distinction marked between erysipelas and adhesive inflammation. The latter being most to the purpose of the work, we shall transcribe.

" Mr. John Bell, in his Discourse upon Wounds, would seem to be afraid of telling you the truth in this instance, lest you should make a bad use of it; and therefore he is very anxious to lead you entirely from the idea of inflammation, and tells you, to shew the oppositeness of their natures, that adhesion prevents inflammation; and that you must only view it on the same footing with the healthy action of vessels, in forming or supporting any part of the system.

" Now that such is not a just representation of the process of adhesson, is evident upon the enumeration of facts. We who know that the natural actions of vessels are analogous to inflammation, might indeed be allowed the liberty of such a comparison; but if we view inflammation perfectly distinct as Mr. Bell does, and say that it has no connexion with healthy actions, and no existence but as disease, how can we call the process of adhesion the same thing as the common actions of health, when we know the symptoms attendant on the adhesive process, are those occasioned by injury and derangement of parts, and which if they were to come on in parts apparently undisturbed and uninjured, would be viewed in the light of disease? Mr. Bell has been very anxious to give you a true idea of the thing, and in his over anxiety to remove a wrong impression with respect to inflammation, he has inadvertently substituted another. It is very true, as Mr. Bell says, adhesion stops inflammation; but in the name of good sense, what procures adhesion, but inflammation? How can Mr. Bell call adhesion an healthy action, when it never takes place but in consequence of disease or injury? Does Mr. Bell call the adhesion of the pleura of the lungs to the pleura of the chest, an healthy action? Are adhesions of the liver healthy functions? On the contrary, whereever we find adhesions after death, do not they bear the direct evidence of some former disease or injury? The adhesion of parts, therefore, is one of those striking instances with which Nature is so bountifully stored, viz. of a dissease setting limits to itself. And to say that the adhesive process is the very opposite of inflammation, is really to deny in the face of facts; for in truth, it never takes place without it.

" Being in possession of the true or relative nature of the action, I think the term Adhesive Inflammation will exactly meet your ideas, and give a just estimation of the process. The adjective " adhesive," will never suffer you to run into the error Mr. Bell

Bell wishes you to avoid, by supposing it of that force which brings on the suppurative process; and the word Inflammation, specified by its title Adhesive, conveys the just state, or true nature, of the process, viz. that the adhesion of parts, or the cure of wounds by what is termed the first intention, can never be effected without a due increased action of the vessels of parts; or, in other words, without the presence of that degree of inflammation, the nature and quantum of which is measured by the term "Adhesive."

"In fact, if the adhesive process were of that delicate and puny nature Mr. Bell represents it, how could deep wounds ever adhere, which are invariably, and indeed necessarily, attended with slight inflammation; for if the parts were not well injected with blood by the increased action, which takes place in consequence of the injury, there could not be a sufficient quantity of lymph thrown out to complete adhesion. And thus we find an evident thickening of parts always attending a severe muscular wound, long after it has healed by the first intention; which is a direct evidence of a certain degree of inflammation having formerly existed.

"A few words only will be necessary to shew you the practical necessity and advantage resting upon a just appreciation of the nature of the adhesive process. And such knowledge is necessarily given by possessing a true sense of inflammation; confining the acceptation and meaning of the word to disease only, is a gross error, and may lead to the grossest abuses. Having, as I trust you now have, a thorough insight into the relative nature of Inflammation, you cannot but view such a misconstruction in the most glaring light. You must feel the absurdity of giving it an independent nature and existence, when it is so evident, on the contrary, that its nature is entirely relative and conditional, when in truth our healthy circulation itself is only inflammation or combustion; such action only becoming disease, when it goes beyond the equilibrium prescribed by the particular economy in which it exists. Nature performs all her curative operations by inflammation; and such action only becomes disease, when it is urged beyond a certain relative boundary or unison of powers; precisely as the mechanical action of rubbing is pleasurable or painful, according to the force employed, and according to the various perceptions of the living surfaces to which it may be applied."

The third Section contains "General Remarks on the Practice of Adhesion." In this the Author shows, that however surgeons may differ in their language, and even in their practice, adhesion must be the ultimate process, and that the object of Surgery should be, to render it as simple as possible. As the inconvenience of sutures is well enough understood in the southern metropolis, which has generally been admitted the best British School of Surgery, we are apt to suppose that the same practice was adopted throughout the kingdom. We should therefore have fancied Mr. Young had been been combating a shadow, had he not so frequently produced Mr. Bell's words, "every wound ought to be sewed." We cannot, however, help thinking, that much less time and trouble would have sufficed to show the fallacy of Mr. Bell's opinions; and perhaps the best way might have been by showing the advantage of leaving Nature to her own resources. Under this impression, we shall dismiss the present article with an extract, containing a more detailed account of the Author's practice.

"The adhesive strap is a convenient slip of firm linen, uniformly covered by a resinous plaster: I say a convenient slip, because the depth and extent of the wound regulate the length of the strap, and its breadth is accordingly proportioned by the length. Without any attempt to fix the limits of the several sizes, one would say, the adhesive strap is from an inch long, and a fifth broad, to perhaps a foot and half in length, and an inch and half in breadth. There can be no fixed rule for the size of the strap, any further than that it should be conveniently proportioned, and that its length should extend considerably beyond the dimensions of the wound on each side.

"The first thing we are naturally led to observe in the adhesive strap is, that, among its several advantages, *it has none of the disadvantages* of the thread suture. It is as obvious that the strap, in drawing the sides of a wound together, can never act as a foreign body to the cavity, as that the thread suture can never be otherwise. It is also as evident that neat slips of plaster, placed in regular succession, and overlapping each other by neat margins, commencing in a sound part, and continuing over the extent of a wound until they again end on a sound part, can no more produce "partial stricture," than the use of the thread suture can avoid it: So far then as it cannot possibly produce "partial stricture," or ever act as "a foreign body," the practice of the adhesive strap is perfectly congenial to the nature of adhesion. The same evidence which demonstrates, that by the adhesive strap "partial stricture" never is incurred, also proves it perfectly compatible in answering the purposes of adhesion, so far as *uniform compressure* is required. The adhesive straps, as a compress to a *wound*, are precisely what the roller is to a *limb*; their application is precisely on the same principle; with this difference, that in the instance of the straps there is more certainty; their marginal overlappings can be more accurately effected; they can be more easily adapted to the surface; and, when applied, their adhesive quality affords a security of equable retention, which cannot be had in the circles of the roller. There is further advantage in the strap, which you never can have in the thread suture; it enables you, from the elastic quality of the integuments, to *replace*, by *extension*, parts that have even been destroyed. See then what you are in possession of! A man gets a desperate pitch among a heap of rough stones; is grazed by the wheel of a heavy cart against a wall; or, in piling up a number of large casks, one of them them falls, and catching his head, a long slip of his scalp is ground away. Here the prospects of adhesion are entirely destroyed; there is a *loss of substance.* Nature can do nothing for herself but by the way of suppuration and granulation; this then is the time to make some return for her many kindnesses. You have it in your power to meet her half way; and by your adhesive straps, you may borrow that portion she has left, by drawing the integuments over the wound till the retorted edges are brought in contact, and retaining them till their adhesion is effected.

"From the nature of the application, you can never have those drags on the sides of a wound with the adhesive straps, which must ever attend the thread suture; at least, so long as its effects are not superseded. This is a circumstance of great consequence in the general practice of procuring adhesion. Observe the oppositeness of the case in the two: when you apply the thread suture, the drag is entirely on the sides of the wound; for you pass your needle at the bottom, through, on each side; and by tying the suture at the top, strain the lips in contact from the general mass; in fact, the sides of a wound, in the application of the thread suture, are in a manner *insolated* from the other parts. Now, in the applications of the strap, it is not the *sides* of the wound you drag; you place your straps according to the extent and depth of the wound, at a considerable distance from its sides; you strain is then applied to a *general sound mass*, which, when drawn upwards or downwards in a cross direction towards the wound, at last includes one of its sides; and, the strap passing over, you bolster up the other side in like manner, by inclosing *some proportion* of the contiguous parts; so that you see in this instance, the *sides* of the wound are the *centre* of your dressings, and, consequently, the most remote from strain, the drag necessarily being at the *points* where your straps commence and end; whilst in that of the thread suture, on the contrary, it is the *sides* of the wound themselves which are dragged from the *general mass*, and not the general mass towards the sides."

The subject is further illustrated by several practical remarks. After this we have a Chapter on the more particular and individual practice of the Thread Suture, (which we thought we had done with) in the reunion of wounds. In this the Author extends his remarks to other writers beside Messrs. Bell, and shows how fallacious and contradictory their doctrines are, and how dangerous their consequent practice proved.

In the fifth Section the Author endeavours to show, that the adhesive strap is competent to the cure of wounds made in the transverse as well as the longitudinal direction of the muscles; and that in bruised wounds and other instances of comminution or loss of substances, in which the suture is not only dangerous but absolutely useless, the adhesive strap will produce an easy cure. —The summary of the whole is contained in a few *concluding* pages.

To

To speak candidly of these pages, we are ready to admit, that as a Course of practical Lectures to uninformed Youth, who may be led astray by *Systems of Surgery*, they might be very useful. To general readers they contain but little information; and that informati n, from the causes above mentioned, much too minute and diffused.

